# Multiproxy bioarchaeological data reveals interplay between growth, diet and population dynamics across the transition to farming in the central Mediterranean

**DOI:** 10.1038/s41598-023-49406-5

**Published:** 2023-12-11

**Authors:** E. W. Parkinson, S. Stoddart, V. Sparacello, F. Bertoldi, O. Fonzo, C. Malone, E. Marini, F. Martinet, J. Moggi-Cecchi, E. Pacciani, L. Raiteri, J. T. Stock

**Affiliations:** 1 Archaeology & Palaeoecology, Queen’s University Belfast, Belfast, UK; 2 Department of Archaeology, University of Cambridge, Cambridge, UK; 3 Dipartimento di Scienze della vita e dell’ambiente, Università degli Studi di Cagliari, Cagliari, Italy; 4 Dipartimento di Studi Umanistici, Università Ca Foscari Venezia, Venice, Italy; 5 Museo Archeologico “Genna Maria” di Villanovaforru, Villanovaforru, Italy; 6 Soprintendenza per i beni e le Attività Culturali della Valle d’Aosta, Aosta, Italy; 7 Dipartimento Di Biologia, Università degli Studi Firenze, Florence, Italy; 8 Soprintendenza Archeologia, Belle Arti e Paesaggio di Firenze, Pistoia e Prato, Florence, Italy; 9 Department of Anthropology, Western University, London, Canada

**Keywords:** Anthropology, Archaeology

## Abstract

The transition to farming brought on a series of important changes in human society, lifestyle, diet and health. The human bioarchaeology of the agricultural transition has received much attention, however, relatively few studies have directly tested the interrelationship between individual lifestyle factors and their implications for understanding life history changes among the first farmers. We investigate the interplay between skeletal growth, diet, physical activity and population size across 30,000 years in the central Mediterranean through a ‘big data’ cross-analysis of osteological data related to stature (*n* = 361), body mass (*n* = 334) and long bone biomechanics (*n* = 481), carbon (*δ*^13^C) and nitrogen (*δ*^15^N) stable isotopes (*n* = 1986 human, *n* = 475 animal) and radiocarbon dates (*n* = 5263). We present the observed trends on a continuous timescale in order to avoid grouping our data into assigned ‘time periods’, thus achieving greater resolution and chronological control over our analysis. The results identify important changes in human life history strategies associated with the first farmers, but also highlight the long-term nature of these trends in the millennia either side of the agricultural transition. The integration of these different data is an important step towards disentangling the complex relationship between demography, diet and health, and reconstruct life history changes within a southern European context. We believe the methodological approach adopted here has broader global implications for bioarchaeological studies of human adaptation more generally.

## Introduction

The transition to farming resulted in profound changes in human society, lifestyle, diet and health^1^. Today, almost all of the world’s population relies on agriculture, demonstrating just how fundamental this process was. While a series of studies have hinted at bio-cultural interactions between diet, growth, health, and demography at the transition to agriculture^2,3^, this has not been explicitly tested. This is largely due to the challenges of exploring the interplay between these factors in prehistoric contexts, given the fragmented nature of the archaeological record and the complex relationship between the biological processes at play. However, a growing body of work is now beginning to demonstrate the potential of cross-analyzing bioarchaeological data and associated radiocarbon dates from single individuals in order to examine temporal trends in diet or body size on a continuous timescale^2,4^, or interrelations between diet, climate and demography^5,6^. In this paper, we investigate long-term trends in skeletal growth, lifestyle, diet and population dynamics in Mediterranean Europe through analysis of complementary bioarchaeological and archaeological data in order to reconstruct life history changes among early farming communities.

From its beginnings in the Near East, agriculture spread into Europe from 9000 to 6000 years ago through two main routes: a northern terrestrial route into northern-central Europe, and a rapid southern maritime route which followed the northern coast of the Mediterranean Sea^7,8^. The central Mediterranean, comprising the Italian peninsula and its surrounding islands, is among the most important regions for understanding this process in southern Europe. Our understanding of the spread of farming in central-western Mediterranean Europe has been much less detailed than that of other regions in temperate Europe, where large bodies of chronological and bioarchaeological data are more widely available^9,10^. However, recent research programs have begun to rapidly alter this situation^11–13^. Although the primary focus of our study is on early farming societies, we also include comparative data from pre-agricultural and historical groups in order to frame our results and discussion within the broader context of long-term trends across the late Pleistocene and Holocene. Whilst our study is representative of the existing body of human bioarchaeological data for the region spanning the last 30,000 years, there are gaps in the earlier (25,000–15,000 BP) and later (3000–2500 BP) aspects of our chronological focus (see Supplementary Information, Sect. 1).

### Archaeological record

The central Mediterranean (Figure S1) is among the most archaeologically rich and well-studied regions in the world, with evidence spanning from the first peopling of the European continent^14^ through to the emergence of complex social, cultural and political structures during the classical and medieval periods that laid the foundations for much of modern western society^15^. As with many regions, the emergence of agriculture was the catalyst for these later developments, and the central Mediterranean played a crucial role in the rapid spread of farming throughout southern Europe^16^. The earliest farming settlements in central-western Mediterranean Europe are found along the south-east coast of Italy, dating to as early as 8100 BP^17,18^. From there, the first pioneer farmers rapidly spread westward along coastal maritime routes of the Tyrrhenian Sea, reaching the north-west Italian region of Liguria by as early as 7950 BP^19^ and the Atlantic coast of south-west Iberia by at least 7500 BP^20^. By contrast, the arrival of farming in central and northern Adriatic Italy took place several centuries later via a more muted process that was closely intertwined with that of the western Balkan coast^21^. Within a few centuries, by approximately 7500 BP, farming subsistence was fully established across the entire central Mediterranean region^22^. As in wider Europe^23,24^, early farming in the central Mediterranean was characterized by the introduction of mixed agriculture based on traditional “Neolithic package” domesticated plants and animals, pottery technology, new belief systems and the emergence of large sedentary villages^17,25,26^.

### The human bioarchaeology of early farmers

The transition to agriculture is a major evolutionary milestone that has driven selective pressures and human adaptation over the last 12,000 years of the human story. The advent of aDNA has especially helped in identifying evidence for selection and associated adaptation events^27^. The human bioarchaeology of this process has received much attention, having been outlined in classic studies^28–30^, as well as more recent reassessments^3,31–33^. In general, the shift to a farming lifestyle is associated with a population ‘boom’ and a series of negative health impacts as communities changed their diets, became sedentary and gathered into larger settlements with poorer sanitation and closer contact with animals, leading to an increase in infectious and zoonotic diseases^34–36^. A number of studies have observed stark declines in body size across the transition to agriculture in some regions^37–39^, pronounced changes in physical activity^40–45^, sharp dietary shifts^46^ and an increase in skeletal signs of stress^28,47^. However, a growing body of research has also shown that changes in body size^2,39^, physical activity^45,48^, diet^49,50^ and prevalence of infectious diseases^51,52^ between hunter-gatherers and farmers were not as distinct in many regions. These studies have brought to light a mosaic of changes associated with farming across different global contexts and underscore the need for region specific studies.

More recently, the transition to farming has been explicitly reassessed within the framework of life history theory^3,7^. This model argues that the ecological, economic and social transformations associated with the transition to agriculture had a major impact on the reallocation of energy away from biological functions such as growth and maintenance and towards increased immune function and reproductive ability. However, these factors could ultimately trade-off against one another during any point in the past where there is a major shift in energy allocation (i.e., later cycles of demographic boom associated with urbanism). Where such approaches have been applied, they have revealed important insights into lifeways of early farmers and the intersection between biological adaptation and culture^2,38^, and are particularly informative when viewed across the *longue durée*^2,4–6^. Directly relevant to the archaeological and regional context of our research are a series of studies that have explored development growth and early life conditions among the first farmers in north-western Italy. These studies provide credible evidence for an energetic trade-off towards elevated immune function at the expense of growth among early agriculturalists in Europe^53–55^. When considered together, these studies highlight the value of large-scale multiproxy approaches that seek to disentangle the picture of human adaptation across the transition to farming at the regional level. Advancements in archaeological science and multi-disciplinary ‘big data’ approaches also make it possible to efficiently draw together and cross-analyze multiple strands of bioarchaeological evidence, enabling new and detailed understandings of past lifeways and life history transitions.

## Results

We use four proxies to explore the long-term interplay between body size and skeletal growth, physical activity, diet and population size, including; (1) estimations of body size, as represented by stature (cm) and body mass (kg), derived from direct measurements of the femur to explore skeletal growth and development, (2) reconstructions of physical activity using long bone cross-sectional geometry (CSG), which models the long bones has structural beams in order to understand their mechanical properties, (3) analysis of *δ*^13^C and *δ*^15^N stable isotopes in order to reconstruct broad patterns of dietary change and consumption, and (4) analysis of a large dataset of radiocarbon dates in order to model population dynamics. We also include additional analysis in our Supplementary Information file.

### Skeletal estimations of body size

**Figure 1:**
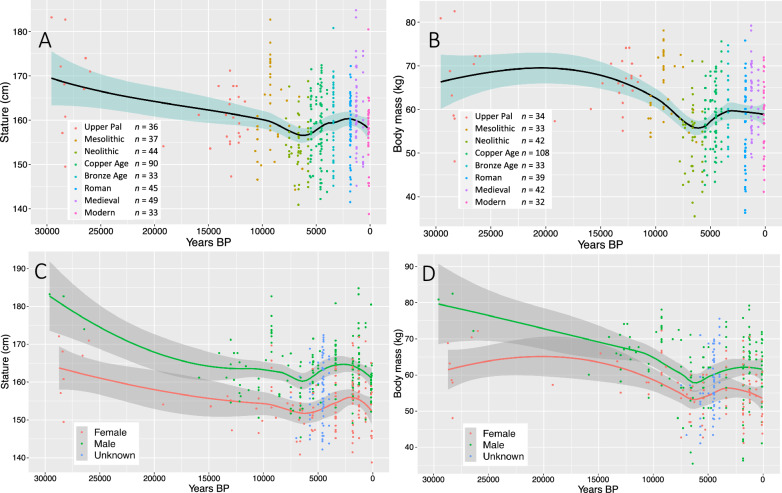
Temporal trends in stature (**A**, *n* = 361) and body mass (**B**, *n* = 334) across 30,000 years. Temporal trends in stature (**C**) and body mass (**D**) by sex (data points in blue represent individuals of unknown biological sex).

Estimated stature (cm) and body mass (kg) were collected from 383 individuals spanning 30,000 years before present (Table S2). The results show a progressive long-term decline in mean stature throughout the late Pleistocene and into the early-mid Holocene until around 9000 BP when a sharper decline occurs and mean stature reaches its minimum around 6500 BP (Fig. 1, Table S3). Mean body mass follows a broadly similar pattern of decline, although initiating later around 20,000 BP and also reaching a minimum at 6500 BP (Fig. 1, Table S4). Both stature and body mass gradually recover in the millennia following 6500 BP. These trends are broadly apparent in both males and females (Fig. 1C-D), although body size appears to be more stable throughout time among females compared to males (Table S5-S8). Our LOESS plots (Fig. 1D) and boxplots (Figure S5-S6) also detect a notable lag in recovery in mean stature among women in from 6500 to 2000 BP.

### Cross-sectional geometry (CSG) of the upper limb

**Figure 2:**
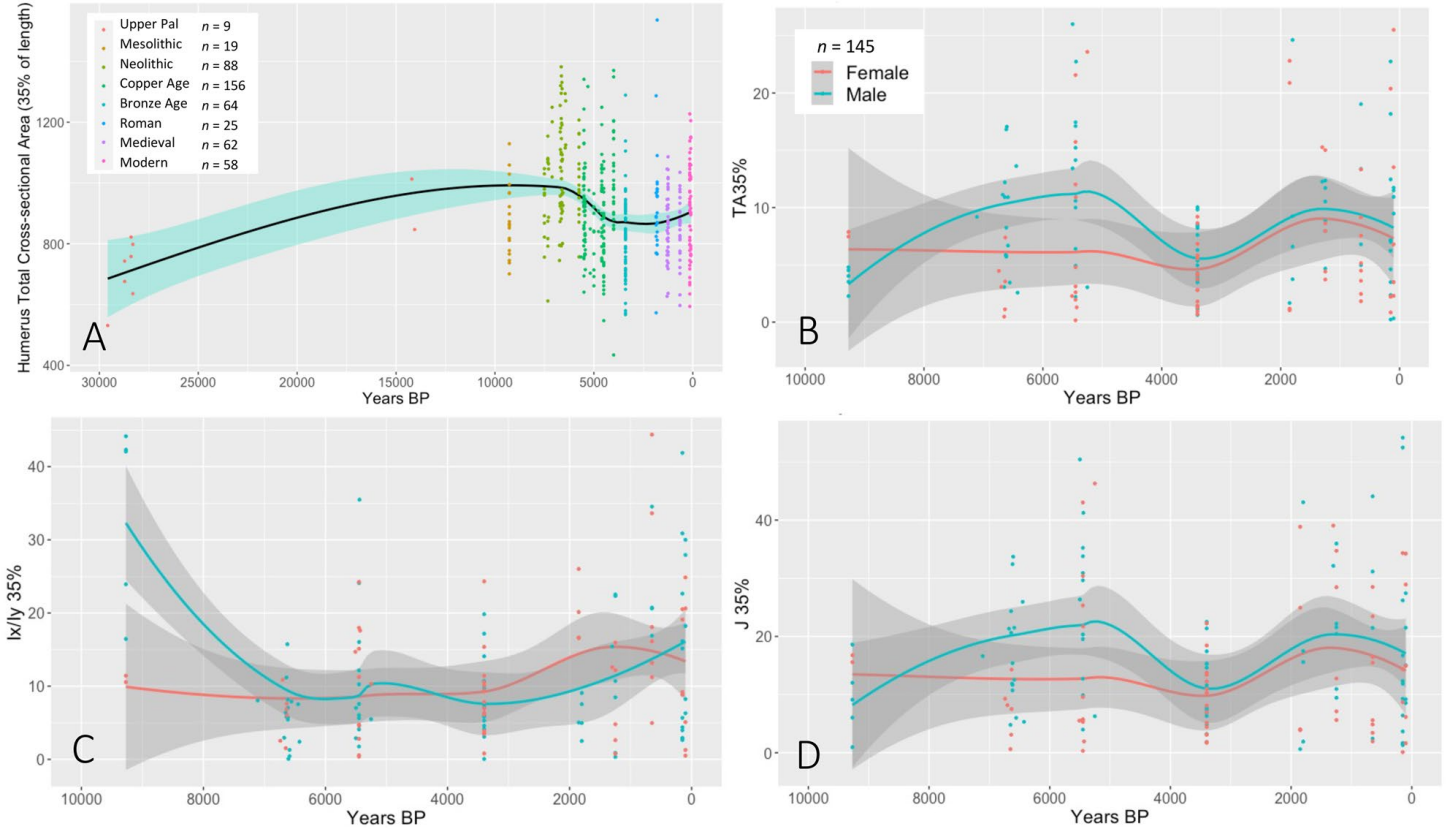
(**A**) Total cross-sectional area of the mid-distal humerus (TA 35%), (**B**) Asymmetry (%AA) in cross-sectional area of the mid-distal humerus (TA 35%), (**C**) Asymmetry (%AA) in cross-sectional shape of the mid-distal humerus (*I*_*x*_/*I*_*y*_ 35%), (**D**) Asymmetry (%AA) in bending rigidity of the mid-distal humerus (*J* 35%).

Cross-sectional geometric (CSG) properties of 481 humeri were used to measure residual bone strength as a means of reconstructing patterns of physical activity in the upper limb^56^, with caveats (see methods). Total cross-sectional area of the mid-distal humerus (TA 35%) was used to explore broad patterns of upper limb robusticity over time (Fig. 2, Table S9). The limited data available indicates an increase in TA 35% across the terminal Pleistocene, followed by relative stability across the early Holocene until a sharp decline from 6500 to 4000 BP. Absolute Asymmetry (%AA) was also used to explore asymmetry in upper limb cross-sectional shape (*I*_*x*_*/I*_*y*_) and robusticity (*J* and TA) in a subset of 145 well preserved individuals (Table S10). The results showed that asymmetry in humeral properties was stable across the Holocene among females, in contrast to males. Heightened asymmetry is particularly apparent in upper limb cross-sectional area (TA 35%) and bending rigidity (*J* 35%) among males between 6500 and 5500 BP (Fig. 2), and signal greater divergence between activities between males and females after the arrival of farming.

### ***δ***^13^C and ***δ***^15^N dietary stable isotopes

**Figure 3:**
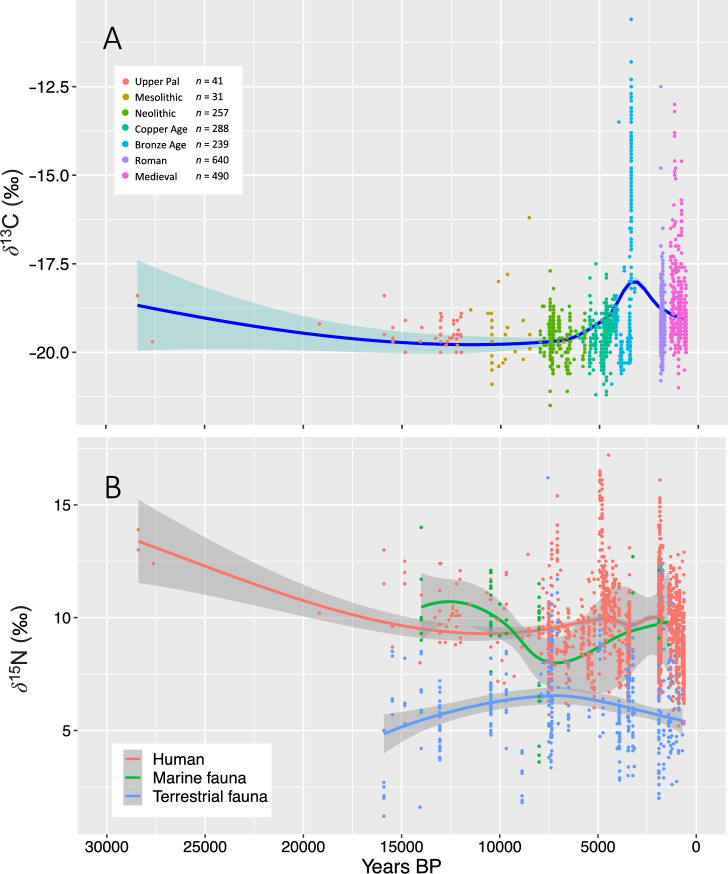
(**A**) Temporal trends in *δ*^13^C in 1986 human individuals from the central Mediterranean (see Fig. S1), (**B**) Temporal trends in *δ*^15^N in humans (*n* = 1986), terrestrial (*n* = 409) and marine fauna (*n* = 66).

*δ*^13^C and *δ*^15^N stable isotopes for 1986 human individuals spanning 30,000–400 BP were gathered from published sources (Table 1; Figure S11) to explore temporal trends in diet. *δ*^15^N is perhaps most informative for exploring dietary trends across the study period (Fig. 3B). Isotope data for humans in Fig. 3B was viewed against a substantial baseline dataset (*n* = 475) of terrestrial and marine fauna. The results show a progressive decline in *δ*^15^N enrichment across the Pleistocene leading into stability across much of the Holocene. Between 4000 and 2400 BP *δ*^15^N enrichment among the samples declines but with a noticeable uptick in *δ*^13^C (Fig. 3A), although some of these trends may be partly influenced by data aggregation and a lack of stable isotopes for the period spanning 3500–2000 BP. *δ*^15^N signals apparent the marine and terrestrial faunal baselines also indicate long-term fluctuations across the terminal Pleistocene and Holocene that do not appear to have a major bearing on the trends in human diet (Fig. 3).

**Table 1:** Summary statistics and references for dietary stable isotope data.

Period	*n*	Mean ± s.d. *δ*^13^C (‰)	Mean ± s.d. *δ*^15^N (‰)	References
Upper Palaeolithic	41	− 19.41 ± 0.42	10.56 ± 1.40	^57–66^
Mesolithic	31	− 19.49 ± 0.94	10.01 ± 1.52	^59,60,62,66–70^
Neolithic	257	− 19.56 ± 0.51	9.25 ± 1.48	^70–78^
Copper Age	288	− 19.51 ± 0.58	10.66 ± 2.07	^71,75,79–83^
Bronze Age	239	− 17.94 ± 2.52	8.98 ± 1.26	^11,71,75,84–89^
Roman	640	− 19.08 ± 0.66	10.32 ± 1.63	^90–94^
Medieval	490	− 18.83 ± 1.09	8.97 ± 1.45	^90–93,95–97^

### Radiocarbon inferred population dynamics

**Figure 4:**
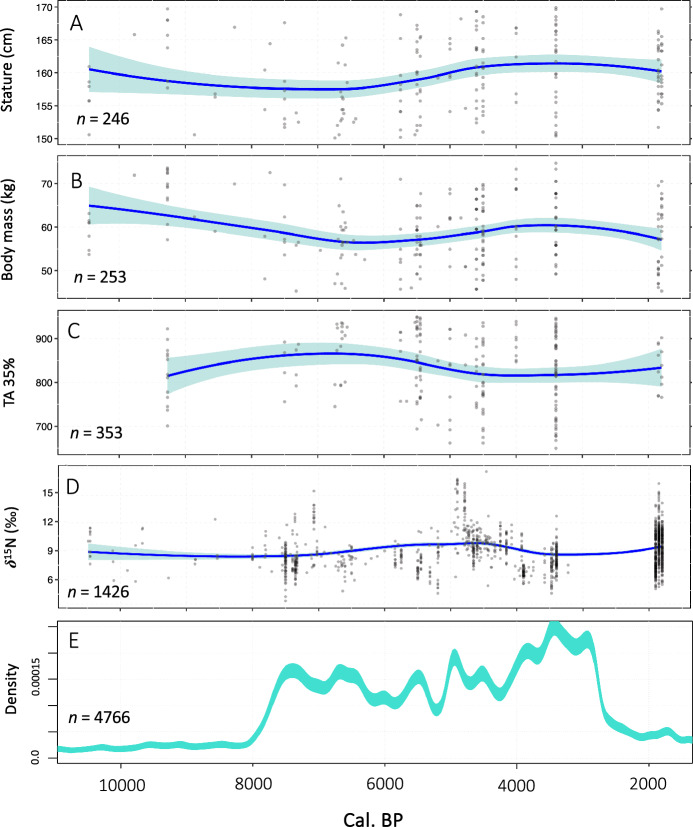
Temporal trends from 10,000 to 2000 years BP in (**A**) estimated stature (cm), (**B**) estimated body mass (kg), (**C**) upper limb robusticity (Total Cross-sectional Area, TA 35%) (**D**) diet (*δ*^15^N), and (**E**) KDE estimated population size derived from radiocarbon dates. Transition to agriculture occurred around 8100 years BP.

We analyzed 5263 radiocarbon dates from 1330 prehistoric archaeological sites in the central Mediterranean (Figure S1) using Kernel Density Estimation (KDE). This methodological approach uses the frequency of occupied sites with dateable material as a proxy for settlement density and demographic change, having been used to reconstruct fluctuations in population size in a range of global contexts^98,99^. The model of demographic change we present in Fig. 4E is restricted to the early-Holocene onwards, with the full span of dates represented in Figure S2. Detailed discussion on trends within individual sub-regions of the central Mediterranean have been provided elsewhere^22^. The KDE model shows a low signal of population activity in the region until a mid-Holocene period growth initiating at 8100 BP, followed by a pronounced peak around 7500 BP (Fig. 4E). Following the 7500 BP peak there is a prolonged period of decline leading to around 6450 BP, after which there are considerable fluctuations and a series abrupt peaks and troughs between 6500 and 2200 BP. Starting at 4200 BP there is further marked growth in the KDE, with the exception of an interruption around 3650 BP, leading to an apex around 3400 BP. Trends following a pronounced drop in the KDE between 2850 and 2650 BP become harder to interpret as the application of radiocarbon dating beyond prehistory becomes less systematic among historical archaeologists.

## Discussion

**Figure 5:**
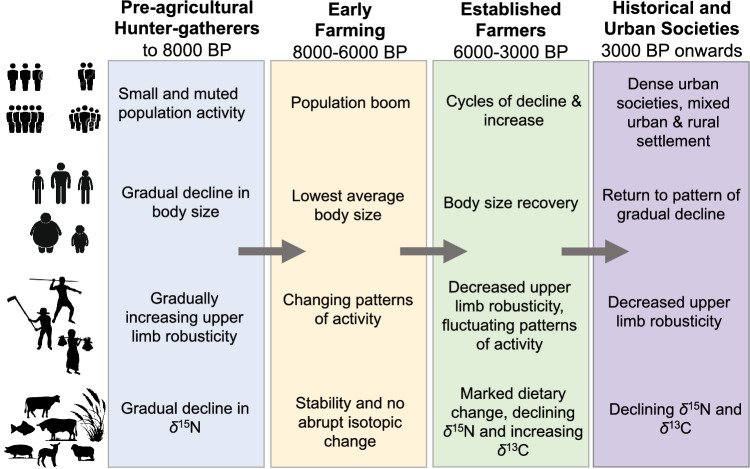
Summary of results of population reconstruction, body size, habitual activity and diet.

This paper investigates the interplay between body size, diet, activity and demography in central Mediterranean prehistory through analysis of supporting bioarchaeological and archaeological data. We have presented the long-term trends identified here on a continuous timescale, in an attempt to step away from grouping our data into cultural groups based on assumed dichotomous time periods. Such an approach allows us to achieve greater resolution and chronological control over our analysis and observations. However, aspects of our analysis and discussion still draw on traditional cultural labels, which continue to have some heuristic value given the restricted focus of our study on the central Mediterranean area. The results indicate a mosaic of trajectories in human diet, demography and body size across the transition to agriculture, but crucially illuminate the long-term context of these trends in the millennia before and after farming (Fig. 5).

Our model of population size derived from radiocarbon evidence indicates a marked demographic increase at the advent of farming around 8100 BP, reaching a peak at 7500 BP. This result tracks the signal of the initial major population boom and increased fertility that is associated with the spread of agriculture and sedentism into Europe^100,101^. Although the Mediterranean region has evidence for comparatively higher densities of pre-agricultural hunter-gatherers^102^, the muted signal in the KDE for the terminal Pleistocene and early Holocene reflects the record of small and dispersed human settlement for Upper Palaeolithic and Mesolithic Europe^102,103^. Studies of aggregated radiocarbon data have already proven to be a particularly useful means of visualizing this phenomenon in wider Europe^99,104^, and a series of focused studies across different sub-regions of the central Mediterranean have independently shown how the of arrival farming was associated with heightened settlement density and significant population growth^22,73,105,106^. Genetic studies have also added to this picture in a global context, estimating a fivefold increase in population growth following the adoption of agriculture in Europe, southeast Asia and sub-Saharan Africa^107^. Archaeological evidence to support the pattern of demographic growth presented here is particularly visible in the south-east of the Italian peninsula where areas of intense nucleated early Neolithic settlement are well-documented^108–110^ and stand as among the densest areas of settlement in Neolithic Europe^111^. The earlier Neolithic in the center and north of the Italian peninsula and central Mediterranean islands was more subdued, but is still associated with an increase in sites and archaeological visibility relative to other periods^112–114^.

The levels of anthropogenic activity in our KDE model throughout later prehistory (4000 BP onwards) demonstrate that the population boom at the onset of agriculture was sustained throughout the Holocene, but not without significant cycles of decline and increase. Settlement evidence and population reconstructions for later prehistory suggest a shift away from the larger villages associated with early farming, towards smaller communities settled within a wider variety of dispersed landscape settings in the Copper Age^115,116^. The emergence of larger conglomerated settlements and proto-urbanism in the region from the middle Bronze Age^117,118^ and into the Iron Age^119^ signals a return to larger habitations and increased population size, before the development of fully urban settlements in the late Iron Age and Roman period onwards. For later time periods, however, radiocarbon data becomes a less powerful tool for attempting to reconstruct population dynamics, but historical documents and better resolved archaeological records allow us elucidate population levels and continued cycles of demographic change throughout the Roman and Medieval periods^120,121^. In a similar vein, a lack of bioarchaeological data for the central Mediterranean for the period 3000–2500 BP (i.e., Iron Age) unfortunately limits our ability to explore changes across the threshold between prehistory and recorded history.

The signal of increased population density and fertility with the arrival of the first farmers provides a useful backdrop with which to consider the results of our analysis of body size, physical activity and diet. Although early farmers are associated with a steep decline in body size, with lowest mean stature and body mass around 6500 BP, the results demonstrate that this took place following a long-term period of gradual decline in body size across the late Pleistocene and into the early/mid Holocene. A subsequent recovery in body size among established farming societies associated with the Copper and Bronze Ages is then followed by a return to a pattern of gradual decline. Interestingly, sharp reductions in body size during the Roman period (ca. 2000 BP) detected in a series of previous studies^67,122,123^ are not present in our LOESS models (Fig. 4), but are somewhat present in our boxplots (Figure S3 and Figure S4). The contrast between these observed trends and two forms of analysis illustrates the influence that grouping of data into traditional archaeological periods can have in obscuring gradual changes in bioarchaeological data over the long-term.

Stature and femoral head size—used to estimate body mass—are the result of a complex process of human growth ^124,125^ influenced by genetics, diet, and other environmental factors^126,127^. The relationship between final adult body size and the environmental context and timing of an individual’s growth during early life is therefore an important means of understanding developmental health in the past. Reduced body size among early farming societies has been documented across a range of global contexts in Europe^38,39,128,129^, North Africa^130^ and North America^30,37,131^, and is widely interpreted in the literature as reflecting a negative impact on skeletal growth stemming from increased physiological stress and decreased dietary diversity^28,30^. The lower body size values for early farmers in our dataset are also suggestive of an adaptive response among early farming societies in the Mediterranean. Heightened risk of exposure to disease during early life among early agriculturalists^132^ could feasibly redirect energy away from skeletal growth towards increased immune function and defense, resulting in overall smaller body size. However, our evidence for demographic growth with the advent farming (Fig. 4E), driven by increased fertility and reproductive ability (decreasing inter-birth intervals), also supports a scenario of life history trade-offs that redirected energy away from skeletal growth towards reproduction^3^.

Recent work by Stock et al.^2^ observed stability in body size in regions of in situ domestication or gradual adoption of agriculture, versus regions where the arrival of agriculture constitutes an abrupt subsistence change in the archaeological record. Absolute chronologies and genetic data appear to show that the first farmers migrated into areas of the central Mediterranean that were unoccupied by hunter-gatherer populations^133–136^, bringing with them the full suite of south-west Asian domesticates. Despite some evidence for acculturation between local hunter-gatherers and farmers (i.e., Sicily and Alpine regions), the arrival of agriculture into the central Mediterranean can be considered a relatively rapid episode of marked subsistence change and significant population turnover. Changes in population structure could be argued to have played some role in the observed changes in body size, as has been suggested elsewhere in the Mediterranean^137^, however a series of studies show discrepancy between genetically predicted height and skeletal estimates of height in prehistoric Europe^138,139^ that underscore the contribution of environmental factors to final adult stature. Current evidence also points to a general picture of population continuity after 8100 BP for much of our study region, with significant episodes of admixture occurring later in prehistory ca. 3500–2900 BP^140–143^, suggesting that changes in population structure did not influence the trends in our data for the mid/late Holocene.

Whilst the early farmers in our dataset represent a period of marked decline in body size, it is important to note that this took place following a longer period of protracted decline initiated in the terminal Pleistocene, and therefore the results cannot solely be interpreted within the context of economic or cultural change. This observation is particularly evident in the analysis of body mass (Fig. 1), and contributes to a growing body of studies that indicate that changes in body size across the transition to agriculture show considerable variation across a range of global contexts^39^, with declines in some cases occurring millennia before the emergence of farming^2^ and forming part of a broader evolutionary trend^144^.

Changes in food supply and procurement, food insecurity and decreased dietary diversity, coupled with the interplay between diet and energy expenditure, have too been argued to have had a major biological impact on early farming communities leading to shifts in life history strategies^3^. The upper limb CSG properties of the individuals analyzed here show changes in patterns of manual activity with the transition to agriculture, including evidence for the emergence of gender specific tasks, in line with broader Europe^145–148^, and a decline in the intensity of manual behavior after 6000 BP. In particular, evidence for increased upper limb asymmetry among early farming males stands in contrast to women, who exhibit consistently low levels of lateralisation and evidence for engaging in bimanual labor. Although it is difficult to attribute our results to specific activity regimes, especially given the highly diverse nature of labor division within agricultural societies^149^, a range of bioarchaeological^40,150^, experimental^148^ and ethnographic^151^ studies support a scenario for women in early farming societies engaging in labor intensive bimanual food processing activities. Interestingly, our data does not show the long-term divergence in male and female patterns of activity that have been reported elsewhere for the later prehistory of southern Europe^145,152^.

Despite the challenges in interpreting the specifics of CSG data, the shift in manual behaviors among early farmers likely resulted in changes to energy expenditure and required food intake^3^. *δ*^13^C and *δ*^15^N stable isotopes offer a way of contextualizing humans within ecological food webs as a means to reconstruct broad dietary patterns^153^, but the factors that impact isotope composition within bone tissue are complex and varied, ranging from the health of the individual^154^, agricultural practices^77,155^ and environmental conditions^156^. A series of now classic studies show a major dietary shift with the transition to agriculture in Atlantic and Baltic regions of North-Western Europe that is characterized by a shift away from consumption of marine protein^46,157^, but no such signal is detected in our data. Instead, our stable isotope data show no significant changes or variation in consumption across the transition to agriculture, indicating a largely terrestrial diet for much of the terminal Pleistocene and early/mid Holocene. Changes in *δ*^13^C and *δ*^15^N enrichment instead occur in our data after 5000–4000 BP.

It is difficult to directly tie stable isotope data to past nutrition^158^, but our results are suggestive of limited change in the nutritional composition of diets between hunter-gatherers and the first farmers of the central Mediterranean. Both palaeodietary studies^49,50^ and faunal assemblages^159^ show that central Mediterranean hunter-gatherer populations appear to have largely exploited terrestrial resources, perhaps due to the lower overall productivity of the Mediterranean Sea^160^. An additional caveat to interpreting differences between Northern and Southern Europe, however, is the variability in nitrogen enrichment between Atlantic and Mediterranean archaeological specimens of fish stemming from ecological differences between both marine contexts^161^. Likewise, the general picture of reliance on terrestrial resources by early farmers has long been confirmed by a series of palaeodietary studies^75,77,78^, even among groups living in coastal regions of southern and northern Italy^74,162^. Archaeological evidence also shows that the early farmers of the central Mediterranean, as might be expected, relied on the typical range of South-West Asian domesticated plants and animals^163–165^, but with regional variation in choice of livestock and crops^166–168^. Whilst some studies have highlighted sub-regional differences in consumption among early farmers^70,78^, stable isotopes do not offer enough resolution to prise apart regional trends apparent in faunal and botanical records. Isotopic evidence for dietary change detected among established farming societies from 5000 BP onwards in our data does, however, reflect a more nuanced picture of prolonged and gradual changes in consumption and human health now emerging from higher resolution analysis of dental calculus^169^. Ultimately, prising apart the implications of long-term reliance on terrestrial resources and the resultant impact on dietary transitions and changes in energetic intake from food is challenging with the methods available at present.

Whilst our palaeodietary data may not paint a picture of a rapid transition, it is clear from the complementary data analyzed here that the onset of agriculture in the central Mediterranean was rapid and irreversible, albeit not without significant cycles of rise and decline (i.e., population size) or periods of continued human adaptation (i.e. body size, physical activity) in the millennia either side of 8000 BP. In spite of the millennial long trends observed here, demographic change appears to have gone hand-in-hand with adaptive changes in body size and regimes of physical activity during the early part Holocene.

## Conclusion

Human skeletal growth and life history are complicated processes, and attempting to untangle the interplay between demography, diet, nutritional status and health is a challenge for bioarchaeologists and evolutionary biologists. Ultimately, any study that seeks to gather together large datasets will face limitations in interpretation and data integration. However, we have attempted a first step towards this within the Mediterranean region by drawing together a range of bioarchaeological proxies to examine the long-term trajectories of human societies across the advent of farming and beyond. The broad picture of human adaptation across the transition to agriculture in the central Mediterranean that we have presented can now be tested and interrogated further through fine-grained analysis of individual sites and sub-regions.

The dietary patterns presented here are one aspect that would welcome further investigation, especially with respect to regional trends and clustering within our dataset (Figure S1B). A lack of available bioarchaeological data for the period between 3500 and 2000 BP also unfortunately limited our ability to explore human adaptation against the backdrop of developing urbanism and state formation during such a dynamic period in later prehistory^12^, and is another potential area for future work. As our study has largely relied on data gathered from published sources, it is not always possible to establish the full contextual details for the individual data points we have collected, thus limiting the extent to which our databases can be cross-referenced. A next obvious step would be to further explore the trends observed here through multi-variate analysis of data for body size, behavior, stable isotopes and radiocarbon dates which can be tied to a single individual, thus enabling investigation of prehistoric life histories and lifeways at the individual scale. We do, however, hope that our results (summarized Fig. 5) will stimulate future bioarchaeological research in the region and wider Europe and encourage others to adopt similar multi-method ‘big data’ approaches that combine multiple strands of bioarchaeological data set on a continuous scale.

## Methods

### Estimation of body mass and stature

Body mass (kg) and stature (cm) were estimated using regression equations developed for southern European Holocene groups using superior-inferior femoral head diameter and maximum length of the femur respectively^170^. Although a combination of femoral and tibial length is usually desired when reconstructing stature from archaeological human remains, the majority of the sites spanning 8000–4000 BP analyzed here are comprised of commingled burial deposits requiring the body size estimations of isolated femora. Comparative body size estimates for the individuals spanning 30,000–8000 BP and 4000–0 BP were derived from raw osteological measurements from published sources^62,171,172^.

### Cross-sectional geometry of the humerus

Cross-sectional geometric (CSG) properties of the humerus correlated with robusticity and rigidity (TA and *J*) and cross-sectional shape (*I*_x_/*I*_y_) were taken at the mid-distal point of the diaphysis (35% of bone length)^56,173^. CSG data spanning the period 8000–4000 BP were derived from high definition 3D models of humeri captured with a NextEngine 3D laser scanner and a DAVID SLS-2 structured light scanner. CSG properties were extracted from the scans using the automated program AsciiSection V3.1^174^. All scan data were processed and aligned to anatomical axes according to standard orientation protocols^175^ in NextEngine 3D Scan Studio and Rapidform XOR. Comparative data from other points in time were derived from published sources^171,172^.

CSG data derived from 3D models are ‘solid’ CSG properties that account for external periosteal contours only, whereas published data were acquired using a method which captures ‘true’ cross-sectional properties comprising of periosteal and endosteal contours^173^. Although ‘solid’ and ‘true’ CSG properties have been shown to be highly correlated^176,177^, they cannot be directly compared. A conservative approach was adopted here to only consider Total Cross-sectional Area (TA 35%). Whilst TA is not the most direct mechanical property for reconstructing behavior, it is used here in order to enable cross-comparison between our data and published sources utilizing the “true CSG” method^171^ and because it is strongly correlated with standard measures of long bone rigidity (i.e. section moduli, polar second moments of area)^56,176,177^. All CSG properties of the humerus were standardized for the influence of body size by powers of bone length following standard protocol^56^. Asymmetry in upper limb CSG properties was investigated using percent Absolute Asymmetry (%AA)^178,179^. Direct comparison of solid and true CSG data to explore temporal trends in upper limb asymmetry is justified here, as %AA can be viewed as representing differences between sides that are relative to each individual and reflects a representative measure of asymmetry irrespective of what method is used.

### Stable Isotope Data

*δ*^13^C and *δ*^15^N stable isotopes for 1986 human individuals (inland, *n* = 1006, coastal *n* = 906) spanning 30,000–400 BP were gathered from published sources (references provided in Table 1). We also created a baseline of terrestrial (*n* = 409) and marine (*n* = 66) faunal *δ*^13^C and *δ*^15^N stable isotope data for the same time span. When compiling our database we did not include any isotopic data that did not have a C:N ratio between 2.9 and 3.6^153,180^. Stable isotope data with missing C:N ratios from Sardinia was originally visually screened by Lai (2008:230–237)^75^ to ensure adherence with aforementioned protocol^180^. Skeletal age, sex, skeletal ID number and contextual information were documented as provided in published sources for all human samples, and faunal samples where relevant. Provided age ranges from human data were consolidated and categorized into broad terms of “adult” and “non-adult” according to published sources.

### KDE analysis of radiocarbon data

A total of 5263 radiocarbon dates from 1330 prehistoric archaeological sites from previously published databases^22,181^ were analyzed using Kernel Density Estimation (KDE) in *R* (V. 4.3.1) using Rowcal^182^. Hierarchical cluster analysis was used to identify unique site phases, which extracts one date from each site phase in order to ensure that over-sampled sites did not skew our model^183^.

### Statistical procedures for bioarchaeological data

We chose to visualize and analyze our data points on a continuous timescale using LOESS plots generated in *R* (V. 4.3.1), with the smoothing span set to 0.85 on all plots (see “Supplementary Information”). Due to sparse bioarchaeological data for the earlier timespan of our study, we grouped individuals into broad categories based on the traditional time periods of Upper Palaeolithic, Mesolithic, Neolithic, Copper Age, Bronze Age, Roman, Medieval and Modern on the basis of the cultural association of each collection and their associated calibrated radiocarbon dates. Each category was analyzed using one-way Analysis of Variance (ANOVA) with Hochberg’s GT2 *post-hoc* tests (see Supplementary Information) performed in SPSS V. 29, which undertake conservative comparisons and greater accuracy when comparing unequal sample sizes^184^.

### Supplementary Information

Supplementary Information.

## Data Availability

The datasets created and analysed for this study are publicly available through Borealis (10.5683/SP3/PYH6SW).
