# Supplementary material for: Multiproxy bioarchaeological data reveals interplay between growth, diet and population dynamics across the transition to farming in the central Mediterranean
Source: Sci Rep. 2023 Dec 11;13:21965. doi: 10.1038/s41598-023-49406-5 (PMC10713518; doi:10.1038/s41598-023-49406-5)
Supplement: Supplementary file 1 — Supplementary Information. [file 41598_2023_49406_MOESM1_ESM.pdf]

Supplementary Information

Multiproxy bioarchaeological data reveals interplay between growth, diet and population dynamics across the transition to farming in the central Mediterranean.

Parkinson, E.W.*[1], Stoddart, S.[2], Sparacello, V.[3], Bertoldi, F.[4], Fonzo, O.[5], Malone, C.[1], Marini, E.[3], Martinet, F.[6], Moggi-Cecchi, J.[7], Pacciani, E.[8], Raiteri, L[6]. & Stock, J. T[9].

[1] *Archaeology & Palaeoecology, Queen's University Belfast, UK*

[2] *Department of Archaeology, University of Cambridge, UK*

[3] *Dipartimento di Scienze della vita e dell'ambiente, Università degli Studi di Cagliari, Italy*

[4] *Dipartimento di Studi Umanistici, Università Ca Foscari Venezia, Italy*

[5] *Museo Archeologico "Genna Maria" di Villanovaforru, Italy*

[6] *Soprintendenza per i beni e le attività culturali della Valle d'Aosta, Italy*

[7] *Dipartimento di Biologia, Università degli Studi Firenze, Italy*

[8] *Soprintendenza Archeologia, Belle Arti e Paesaggio di Firenze, Pistoia e Prato, Italy*

[9] *Department of Anthropology, Western University, Canada*

*Corresponding author: e.parkinson@qub.ac.uk

Table of Contents

Section 1 - Materials 2

Section 2 - KDE analysis of radiocarbon dates 3

Section 3 - Body size 4

Section 3 - CSG 9

Section 4 - Isotopes 13

Section 5 – Statistical Analysis and R Code for LOESS plots 16

References 17

Section 1 - Materials

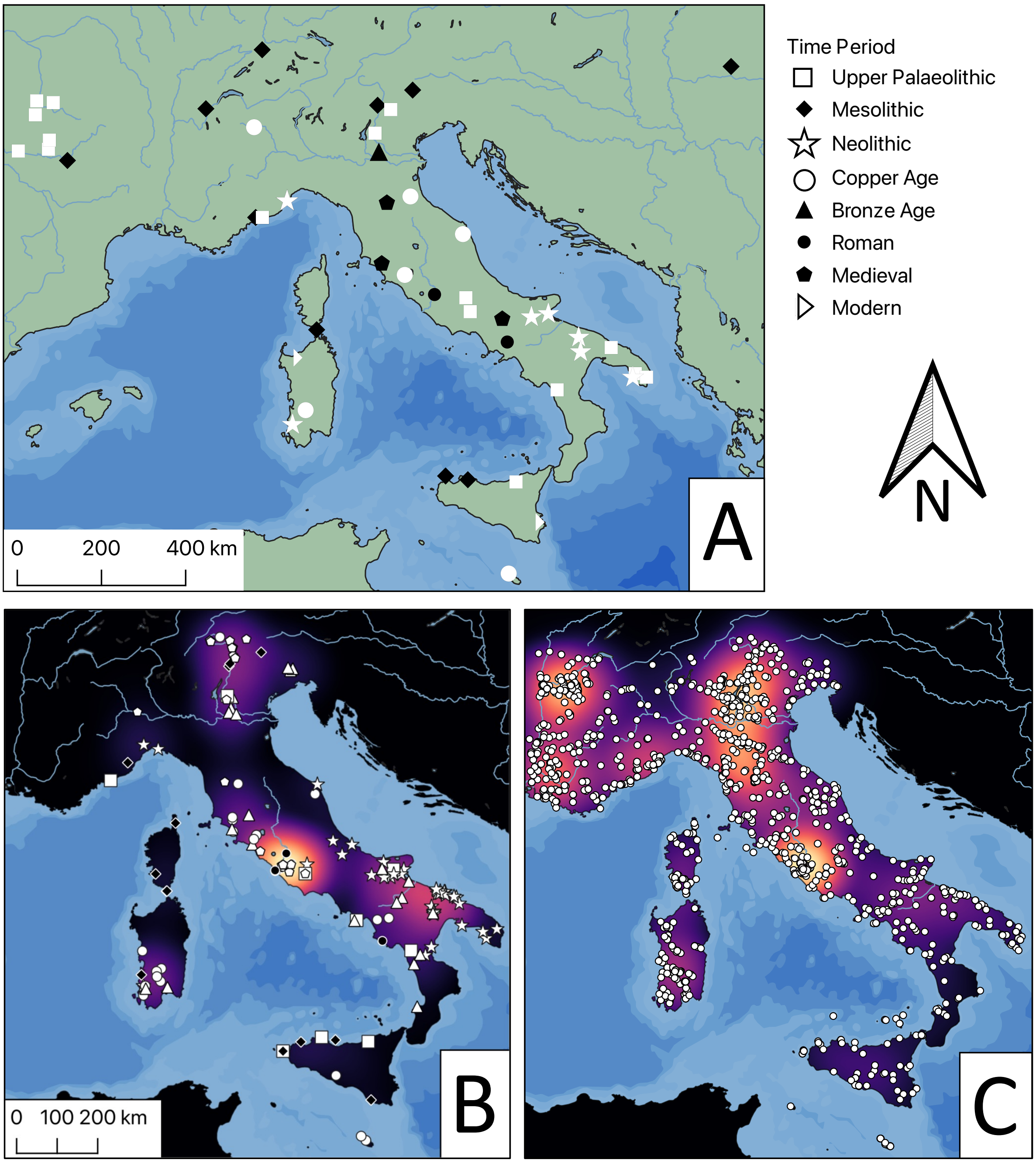

Figure S1: Location map of the central Mediterranean showing the location of sites where the osteometric data (A), stable isotopes (B) and radiocarbon dates (C) used in this study originate from. Full list of site names and coordinates for each dataset are provided in the associated datasets which are publicly available through Borealis repository (DOI: https://doi.org/10.5683/SP3/PYH6SW). Heatmaps for stable isotope (B) and radiocarbon (C) illustrate regional and spatial clustering in the data. References for all published data are included in the main text. All maps were generated in QGIS 3.34.0 (https://www.qgis.org/) using basemaps downloaded from Natural Earth (https://www.naturalearthdata.com/).

Osteometric data relating to stature, body mass and long bone cross-sectional geometry were derived from human skeletons from 56 sites spanning Upper Palaeolithic, Mesolithic, Neolithic, Copper Age, Bronze Age, Roman, Medieval and Modern cultural contexts (Table S1). Data for the Upper Palaeolithic, Mesolithic, Bronze Age, Roman, Medieval and Modern is derived from published sources[1–3], whilst data for the Neolithic and Copper Age was collected independently as part of this study. Owing to the rarity of Upper Palaeolithic and Mesolithic skeletons in the central Mediterranean area, osteometric data for these periods was supplemented by data from individuals from wider Europe (Figure S1a). For the Upper Palaeolithic, individuals from central-southern France were included in the analysis. For the Mesolithic, comparative data from the Iron Gates site of Schela Cladove (Romania) was included. A lack of available bioarchaeological data for the central Mediterranean for the Iron Age (3000-2500 BP) unfortunately limits our ability to explore changes across threshold between prehistory and history.

Table S1: Date range of skeletal materials and their traditional cultural attribution.

Period	Approximate date range of samples
Mid/Late Upper Palaeolithic	32,000-11,500 BP
Mesolithic	11,500-8000 BP
Neolithic	8000-6000 BP
Copper Age	5600-4200 BP
Bronze Age	3500-3100 BP
Roman	1850-1800 BP
Medieval	1250-650 BP
Modern	100-200 BP

Section 2 - KDE analysis of radiocarbon dates

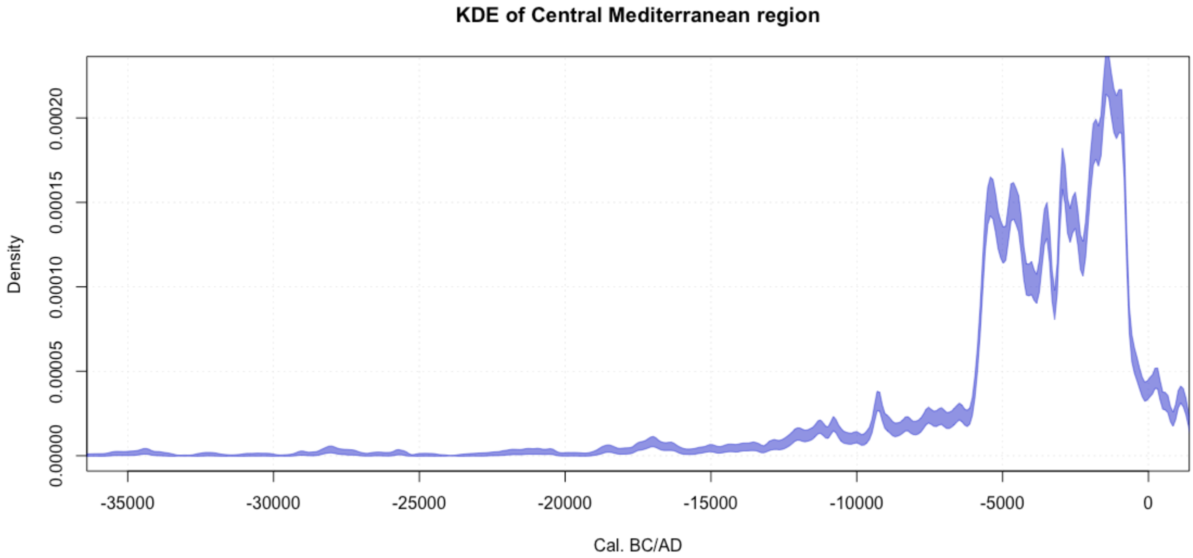

Figure S2: Kernel Density Estimation (KDE) plot of all 5263 radiocarbon dates from 1339 prehistoric archaeological sites in the central Mediterranean spanning the Upper Palaeolithic to Iron Age. Dates for the Holocene[4] and terminal Pleistocene[5] were derived from published literature.

Section 3 - Body size

Table S2: Summary statistics for estimated stature (cm) and body mass (kg) by time period and sex.

	Stature (cm)		Body mass (Kg)	
	n	**Mean ± s.d.**	***n***	**Mean ± s.d.**
**Pooled sex (with disarticulated individuals*)**				
Upper Palaeolithic	36	163.7 ± 9.8	34	66.6 ± 7.1
Mesolithic	37	162.7 ± 9.8	33	65.4 ± 7.4
Neolithic*	44	154.3 ± 6	42	53.6 ± 8
Copper Age*	90	158.5 ± 7.9	108	57.7 ± 7.8
Bronze Age	33	160.2 ± 8.5	33	60.8 ± 7.1
Roman	45	158.3 ± 7.1	39	55 ± 9.9
Medieval	49	162.1 ± 8.9	42	62.6 ± 7.6
Modern	33	157.2 ± 7.7	32	58 ± 8.7
**Males**				
Upper Palaeolithic	22	165.9 ± 8.7	22	68.9 ± 6.1
Mesolithic	24	166.8 ± 8.4	22	67.8 ± 7
Neolithic	17	155.8 ± 5.4	19	54.9 ± 9
Copper Age	19	162.5 ± 4.5	24	58.7 ± 5.7
Bronze Age	17	165.7 ± 6.4	17	64.2 ± 6.9
Roman	23	160.8 ± 5	20	56.4 ± 11.2
Medieval	27	166.6 ± 8.2	25	66.2 ± 6.9
Modern	22	160.4 ± 5.5	22	60.6 ± 8.3
**Females**				
Upper Palaeolithic	14	160.3 ± 8.2	12	62.2 ± 7
Mesolithic	13	155.2 ± 7.7	11	60.5 ± 6
Neolithic	11	151.4 ± 4.7	11	52.7 ± 5.5
Copper Age	11	152.8 ± 5.2	13	52.7 ± 5.8
Bronze Age	16	154.5 ± 6.4	16	57.1 ± 5.4
Roman	22	155.8 ± 8.1	19	53.5 ± 8.2
Medieval	22	156.7 ± 6.5	17	57.3 ± 5.1
Modern	11	151 ± 7.9	10	52.3 ± 6.7

*Contains commingled individuals with no known sex.

Table S3: ANOVA results and Hochberg's GT2 *post hoc* tests comparing stature by time period.

	Upper Pal.	Mesolithic	Neolithic	Copper Age	Bronze Age	Roman	Medieval
Mesolithic	1	-	-	-	-	-	-
Neolithic	<.001	<.001	-	-	-	-	-
Copper Age	0.030	0.195	0.140	-	-	-	-
Bronze Age	0.882	0.998	0.043	0.999	-	-	-
Roman	0.080	0.341	0.430	1.000	0.999	-	-
Medieval	1.000	1.000	<.001	0.280	1.000	0.482	-
Modern	0.026	0.130	0.968	1.000	0.979	1.000	0.193
**ANOVA results**	**df**	**F**	**Sig.**				
	7	6.296448	<.001				

Table S4: ANOVA results and Hochberg's GT2 *post hoc* tests comparing body mass by time period.

	Upper Pal.	Mesolithic	Neolithic	Copper Age	Bronze Age	Roman	Medieval
Mesolithic	1.000	-	-	-	-	-	-
Neolithic	<.001	<.001	-	-	-	-	-
Copper Age	<.001	<.001	0.1113	-	-	-	-
Bronze Age	0.075	0.419	0.003	0.803	-	-	-
Roman	<.001	<.001	1.000	0.839	0.063	-	-
Medieval	0.561	0.982	<.001	0.024	0.999	<.001	-
Modern	<.001	0.007	0.384	1.000	0.994	0.957	0.342
**ANOVA results**	**df**	**F**	**Sig.**				
	7	13.4658	<.001				

Table S5: ANOVA results and Hochberg's GT2 *post hoc* tests comparing stature among males by time period.

	Upper Pal.	Mesolithic	Neolithic	Copper Age	Bronze Age	Roman	Medieval
Mesolithic	1.000	-	-	-	-	-	-
Neolithic	<.001	<.001	-	-	-	-	-
Copper Age	0.960	0.702	0.096	-	-	-	-
Bronze Age	1.000	1.000	<.001	0.992	-	-	-
Roman	0.292	0.081	0.480	1.000	0.506	-	-
Medieval	1.000	1.000	<.001	0.739	1.000	0.085	-
Modern	0.195	0.049	0.660	1.000	0.373	1.000	0.051
**ANOVA results**	**df**	**F**	**Sig.**				
	7	6.523852	<.001				

Table S6: ANOVA results and Hochberg's GT2 *post hoc* tests comparing stature among females by time period.

	Upper Pal.	Mesolithic	Neolithic	Copper Age	Bronze Age	Roman	Medieval
Mesolithic	0.826	-	-	-	-	-	-
Neolithic	0.059	0.995	-	-	-	-	-
Copper Age	0.231	1.000	1.000	-	-	-	-
Bronze Age	0.507	1.000	1.000	1.000	-	-	-
Roman	0.818	1.000	0.926	1.000	1.000	-	-
Medieval	0.976	1.000	0.705	0.981	1.000	1.000	-
Modern	0.039	0.983	1.000	1.000	0.998	0.848	0.576
**ANOVA results**	**df**	**F**	**Sig.**				
	7	2.4166737	0.024				

Table S7: ANOVA results and Hochberg's GT2 *post hoc* tests comparing body mass among males by time period.

	Upper Pal.	Mesolithic	Neolithic	Copper Age	Bronze Age	Roman	Medieval
Mesolithic	1.000	-	-	-	-	-	-
Neolithic	<.001	<.001	-	-	-	-	-
Copper Age	<.001	0.003	0.966	-	-	-	-
Bronze Age	0.766	0.986	0.013	0.512	-	-	-
Roman	<.001	<.001	1.000	1.000	0.070	-	-
Medieval	0.998	1.000	<.001	0.022	1.000	0.001	-
Modern	0.012	0.069	0.419	1.000	0.990	0.876	0.334
**ANOVA results**	**df**	**F**	**Sig.**				
	7	10.049	<.001				

Table S8: ANOVA results and Hochberg's GT2 *post hoc* tests comparing body mass among females by time period.

	Upper Pal.	Mesolithic	Neolithic	Copper Age	Bronze Age	Roman	Medieval
Mesolithic	1.000	-	-	-	-	-	-
Neolithic	0.014	0.119	-	-	-	-	-
Copper Age	0.008	0.083	1.000	-	-	-	-
Bronze Age	0.649	0.993	0.874	0.813	-	-	-
Roman	0.009	0.110	1.000	1.000	0.923	-	-
Medieval	0.682	0.996	0.820	0.744	1.000	0.873	-
Modern	0.012	0.096	1.000	1.000	0.803	1.000	0.740
**ANOVA results**	**df**	**F**	**Sig.**				
	7	4.4583502	<.001				

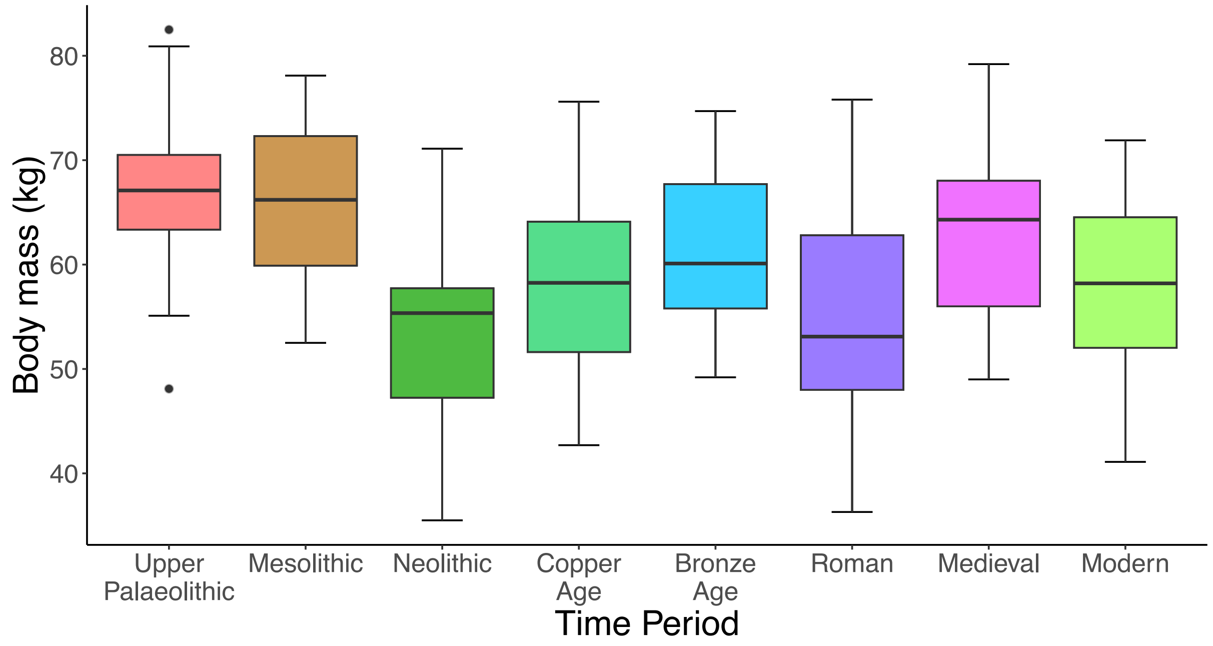

Figure S3: Box plots showing body mass (kg) by time period.

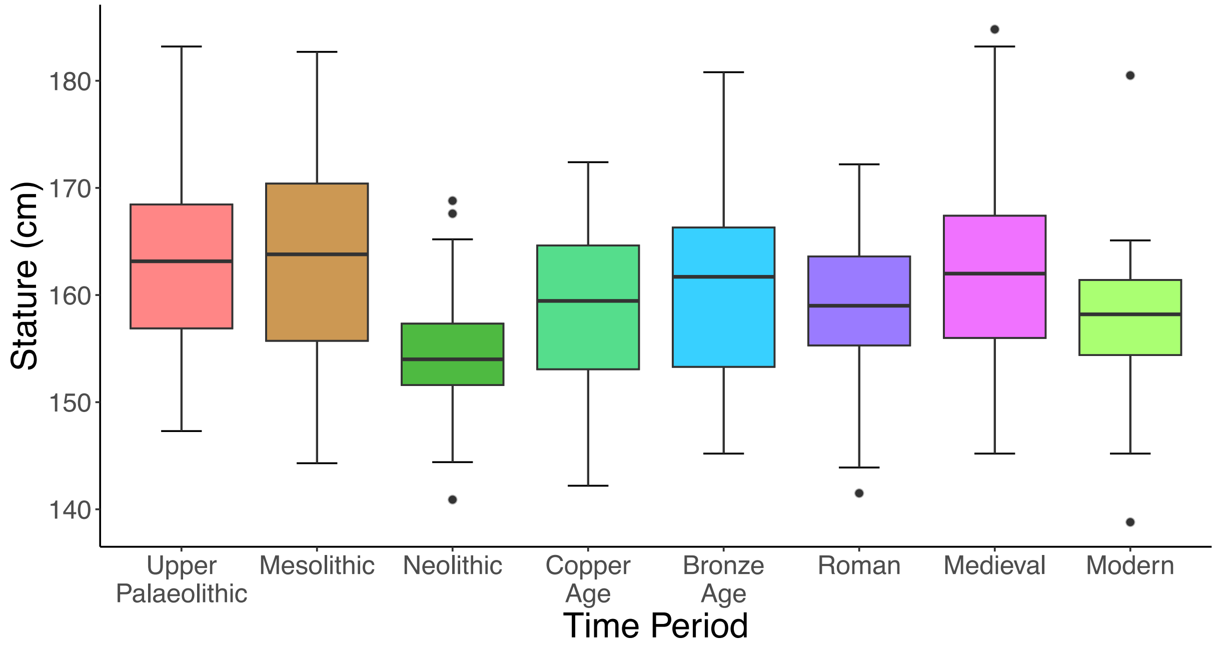

Figure S4: Box plots showing stature (cm) by time period.

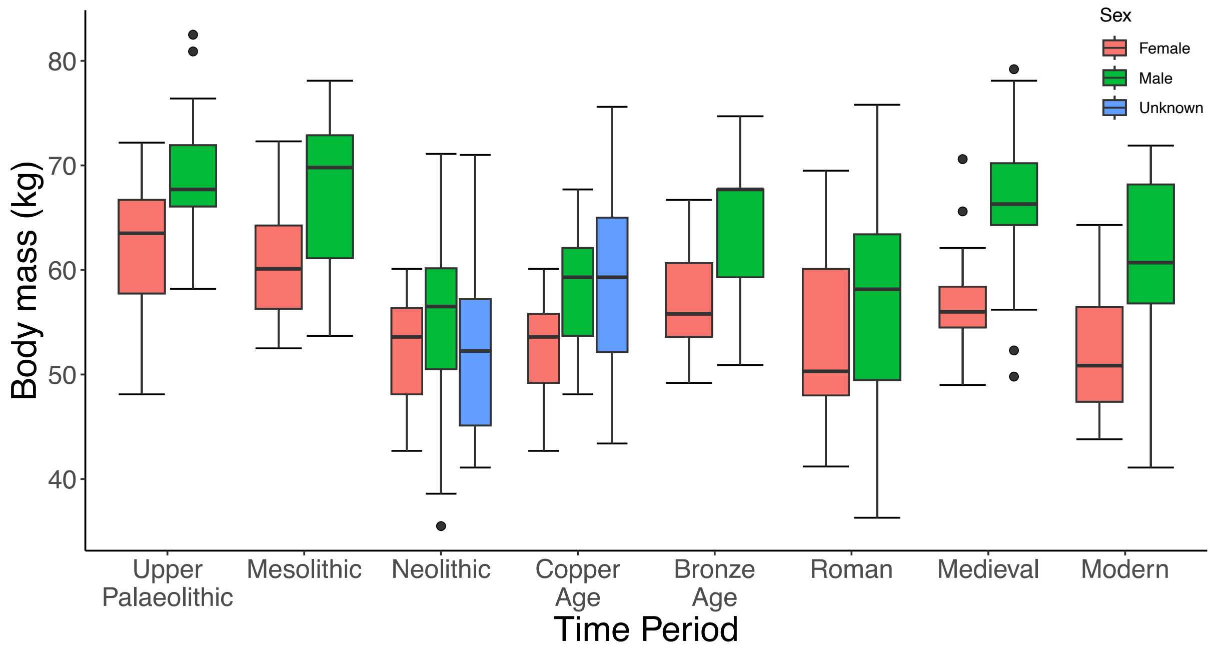

Figure S5: Body mass (kg) by time period and sex.

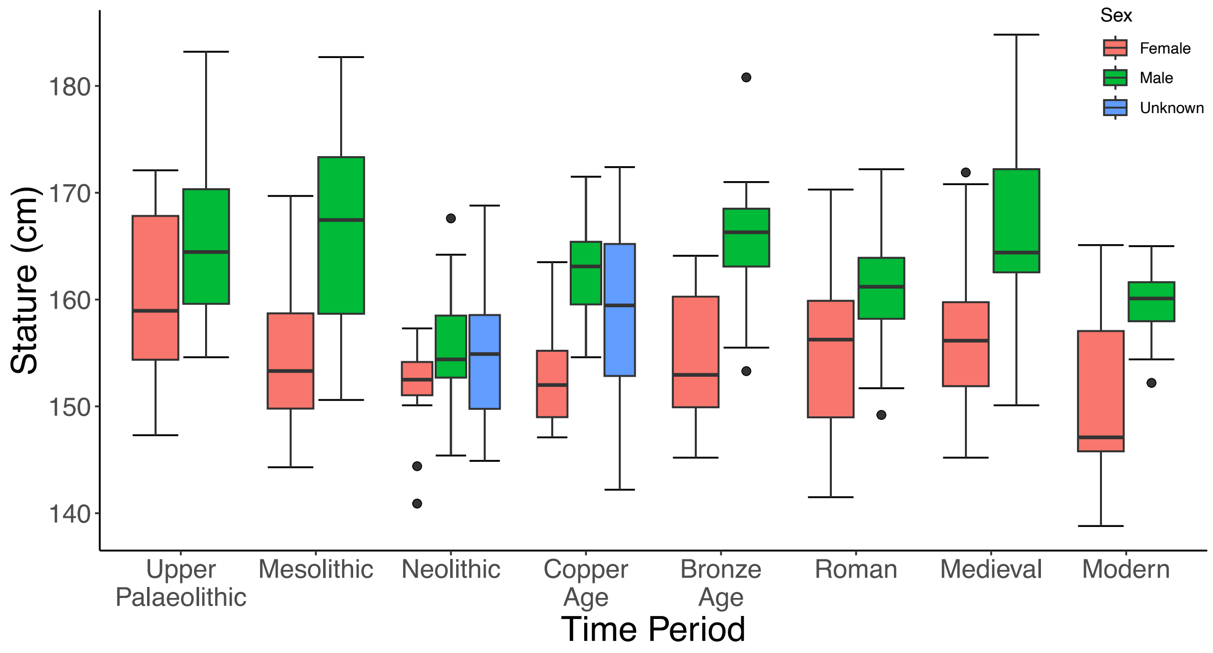

Figure S6: Stature (cm) by time period and sex.

Section 3 - CSG

Table S9: Summary statistics for Total Cross-sectional Area (TA 35%) of the mid-distal humerus diaphysis (35% of bone length).

Time Period	N	Mean	S.D.
Upper Palaeolithic	9	758.22	137.84
Mesolithic	19	882.74	118.02
Neolithic	88	1021.99	159.60
Copper Age	156	911.98	158.29
Bronze Age	64	829.36	134.27
Roman	25	929.68	183.72
Medieval	62	858.77	113.21
Modern	58	912.97	152.77
**Total:**	**481**		

Table S10: Summary statistics for Absolute Asymmetry (%AA) of biomechanical properties of the upper limb (TA 35 = total cross-sectional area, *J* 35 = robusticity, *Ix/Iy* 35% = shape of the mid-distal humerus).

		**TA 35%**		***J* 35%**		**I_x/I_y 35%**	
		Mean	SD	Mean	SD	Mean	SD
**Males**	***N***						
Mesolithic	5	3.83	1.00	9.35	6.60	33.79	12.71
Neolithic	16	8.95	4.57	17.18	9.04	6.01	4.07
Copper Age	13	12.56	7.33	24.64	14.06	10.66	9.60
Bronze Age	16	5.48	3.07	11.16	6.20	8.03	5.21
Roman	5	9.18	9.10	15.74	17.10	5.83	2.54
Medieval	11	10.22	4.19	22.89	12.35	15.78	10.15
Modern	17	8.10	6.12	16.44	16.00	14.63	12.15
**Females**	***N***						
Mesolithic	2	7.67	0.28	16.18	0.83	11.01	0.62
Neolithic	6	3.34	2.48	7.17	4.82	5.90	3.44
Copper Age	12	8.33	8.25	16.74	16.20	10.36	7.77
Bronze Age	15	3.99	2.73	9.06	6.19	8.64	5.71
Roman	4	11.47	12.00	17.93	17.09	19.85	4.44
Medieval	14	7.32	4.52	16.25	12.51	14.56	11.81
Modern	9	8.59	9.08	15.93	13.45	12.23	9.18
**Total:**	145						

Table S11: ANOVA results and Hochberg's GT2 *post hoc* tests comparing Total Cross-sectional Area of the mid-distal humerus (TA 35%) diaphysis by time period.

	Upper Pal.	Mesolithic	Neolithic	Copper Age	Bronze Age	Roman	Medieval
Mesolithic	0.676	-	-	-	-	-	-
Neolithic	<.001	0.007	-	-	-	-	-
Copper Age	0.076	1.000	<.001	-	-	-	-
Bronze Age	0.996	0.994	<.001	0.006	-	-	-
Roman	0.088	1.000	0.169	1.000	0.121	-	-
Medieval	0.817	1.000	<.001	0.397	1.000	0.725	-
Modern	0.106	1.000	<.001	1.000	0.058	1.000	0.74
**ANOVA results**	**df**	**F**	**Sig.**				
	7	12.247	<.001				

Table S12: ANOVA results and Hochberg's GT2 *post hoc* tests comparing AA% TA35% of the mid-distal humerus diaphysis by time among males.

	Mesolithic	Neolithic	Copper Age	Bronze Age	Roman	Medieval
Neolithic	0.008	-	-	-	-	-
Copper Age	0.014	0.715	-	-	-	-
Bronze Age	0.527	0.192	0.061	-	-	-
Roman	0.824	1.000	0.983	0.957	-	-
Medieval	0.006	0.988	0.954	0.062	1.000	-
Modern	0.138	0.999	0.580	0.701	1.000	0.926
**ANOVA results**	**df**	**F**	**Sig.**			
	6	2.923	0.013			

Table S13: ANOVA results and Hochberg's GT2 *post hoc* tests comparing %AA J 35% of the mid-distal humerus diaphysis by time among males.

	Mesolithic	Neolithic	Copper Age	Bronze Age	Roman	Medieval
Neolithic	0.008	-	-	-	-	-
Copper Age	0.014	0.715	-	-	-	-
Bronze Age	0.527	0.192	0.061	-	-	-
Roman	0.824	1.000	0.983	0.957	-	-
Medieval	0.006	0.988	0.954	0.062	1.000	-
Modern	0.138	0.999	0.580	0.701	1.000	0.926
**ANOVA results**	**df**	**F**	**Sig.**			
	6	2.261	0.046			

Table S14: ANOVA results and Hochberg's GT2 *post hoc* tests comparing %AA in I_x/I_y of the mid-distal humerus diaphysis by time among males.

	Mesolithic	Neolithic	Copper Age	Bronze Age	Roman	Medieval
Neolithic	0.050	-	-	-	-	-
Copper Age	0.085	0.667	-	-	-	-
Bronze Age	0.064	0.881	0.97	-	-	-
Roman	0.049	1.000	0.644	0.854	-	-
Medieval	0.211	0.106	0.862	0.297	0.102	-
Modern	0.170	0.133	0.950	0.413	0.128	1.000
**ANOVA results**	**df**	**F**	**Sig.**			
	6	8.019	<0.001			

Table S15: ANOVA results and Hochberg's GT2 post-hoc tests comparing AA% TA35% of the mid-distal humerus diaphysis by time among females.

	Mesolithic	Neolithic	Copper Age	Bronze Age	Roman	Medieval
Neolithic	0.058	-	-	-	-	-
Copper Age	1.000	0.495	-	-	-	-
Bronze Age	0.002	0.998	0.601	-	-	-
Roman	0.989	0.809	0.998	0.847	-	-
Medieval	1.000	0.213	1.000	0.255	0.986	-
Modern	1.000	0.663	1.000	0.751	0.999	1.000
**ANOVA results**	**df**	**F**	**Sig.**			
	6	1.388	0.236			

Table S16: ANOVA results and Hochberg's GT2 post-hoc tests comparing %AA *J* 35% of the mid-distal humerus diaphysis by time among females.

	Mesolithic	Neolithic	Copper Age	Bronze Age	Roman	Medieval
Neolithic	0.044	-	-	-	-	-
Copper Age	1.000	0.519	-	-	-	-
Bronze Age	0.012	0.986	0.711	-	-	-
Roman	1.000	0.852	1.000	0.92	-	-
Medieval	1.000	0.278	1.000	0.48	1.000	-
Modern	1.000	0.580	1.000	0.769	1.000	1.000
**ANOVA results**	**df**	**F**	**Sig.**			
	6	1.054	0.401			

Table S17: ANOVA results and Hochberg's GT2 post-hoc tests comparing %AA in I_x/I_y of the mid-distal humerus diaphysis by time among females.

	Mesolithic	Neolithic	Copper Age	Bronze Age	Roman	Medieval
Neolithic	-	-	-	-	-	-
Copper Age	1.000	-	-	-	-	-
Bronze Age	1.000	0.998	-	-	-	-
Roman	1.000	1.000	1.000	-	-	-
Medieval	0.990	0.200	0.630	0.311	-	-
Modern	1.000	0.502	0.985	0.679	0.997	-
Modern	1.000	0.953	1.000	0.999	0.925	1.000
**ANOVA results**	**df**	**F**	**Sig.**			
	6	1.82	0.112			

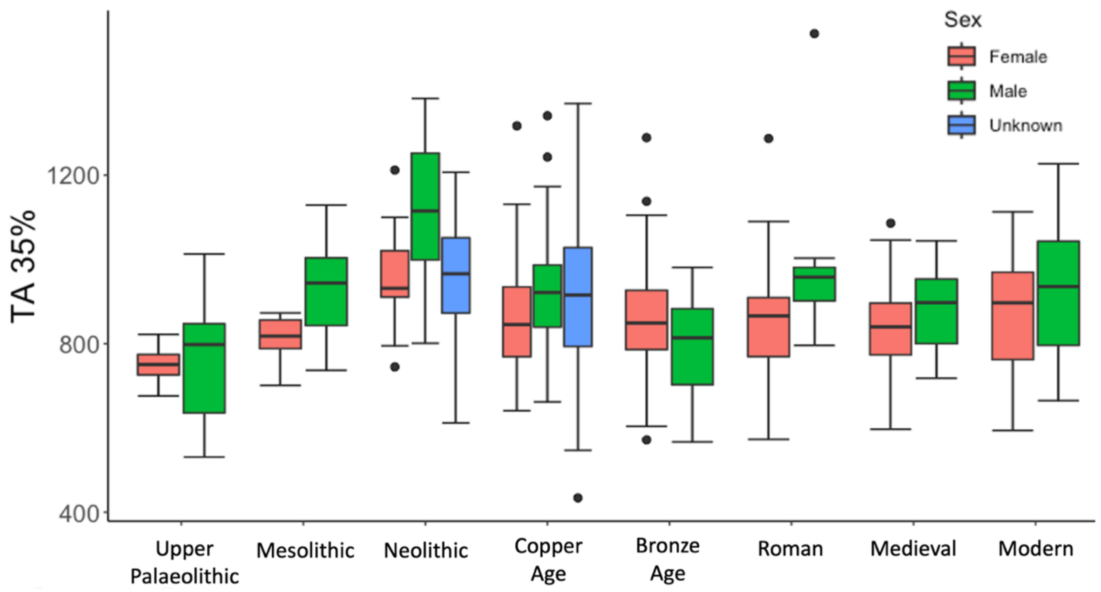

Figure S7: Total cross-sectional area of the mid-distal humerus (35% of bone length) by time period.

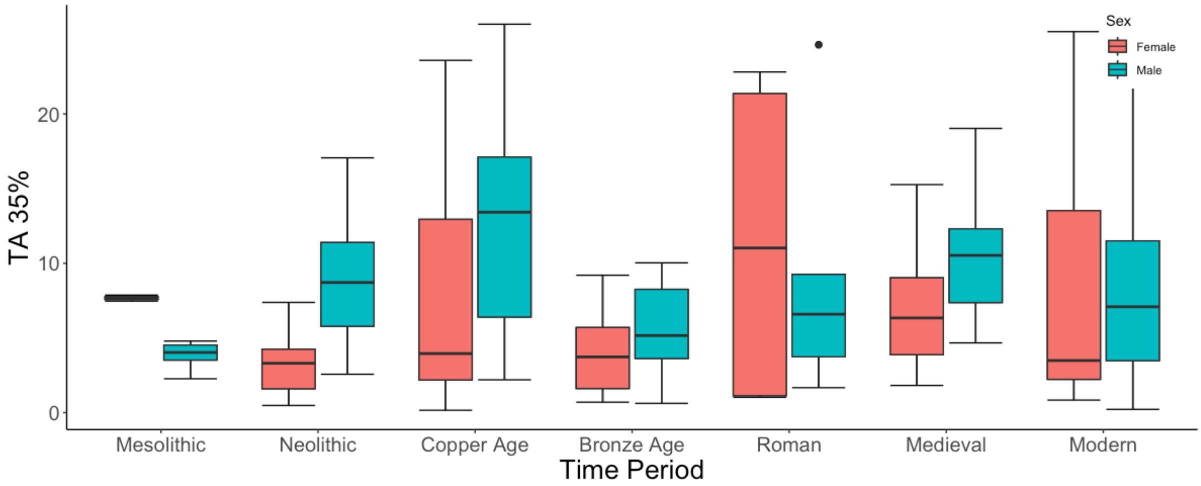

Figure S8: Asymmetry (AA%) in TA (35%) of the humerus by time period.

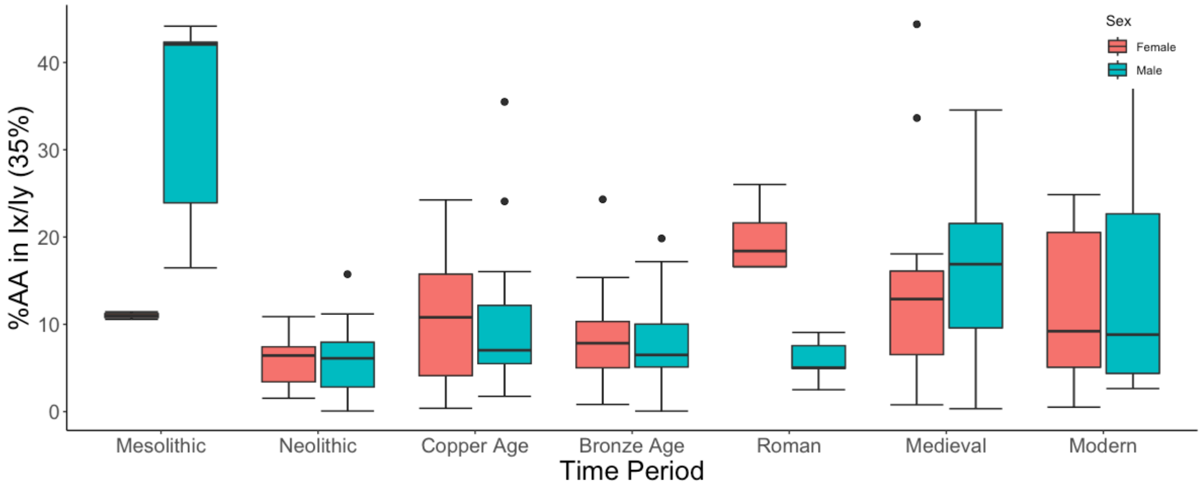

Figure S9: Asymmetry of cross-sectional shape of the humerus (I_x/I_y 35%) by time period.

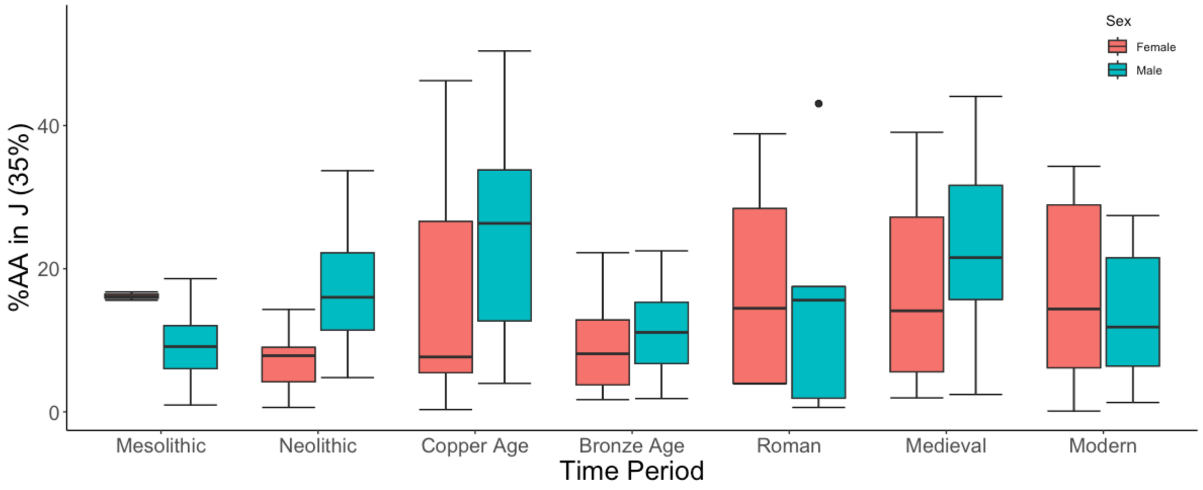

Figure S10: Asymmetry in bending rigidity (*J* 35%) of the humerus by time period.

Section 4 - Isotopes

Table S18: Summary statistics for nitrogen (δ^{15}N) and carbon (δ^{13}C) stable isotopes (see Figure S11).

**Time Period**	***N***	**N13**		**C13**	
		Mean	SD	Mean	SD
Upper Pal.	41	10.56	1.40	-19.41	0.42
Mesolithic	31	10.01	1.52	-19.49	0.94
Neolithic	257	9.25	1.48	-19.56	0.51
Copper Age	288	10.66	2.07	-19.51	0.58
Bronze Age	239	8.98	1.26	-17.94	2.52
Roman	640	10.32	1.63	-19.08	0.66
Medieval	490	8.97	1.45	-18.83	1.09
**Total:**	1986				

Table S19: ANOVA results and Hochberg's GT2 *post hoc* tests comparing Nitrogen 15 (δ^{15}N) by time period.

	Upper Pal.	Mesolithic	Neolithic	Copper Age	Bronze Age	Roman
Mesolithic	0.967		-	-	-	-
Neolithic	<.001	0.228	-	-	-	-
Copper Age	1.000	0.472	<.001	-	-	-
Bronze Age	<.001	0.015	0.709	<.001	-	-
Roman	1.000	0.999	<.001	0.046	<.001	-
Medieval	<.001	0.009	0.362	<.001	1.000	<.001
**ANOVA results**	df	F	Sig.			
	6	64.434	<.001			

Table S20: ANOVA results and Hochberg's GT2 *post hoc* tests comparing Carbon 13 ($\delta^{13}C$) by time period.

	Upper Pal.	Mesolithic	Neolithic	Copper Age	Bronze Age	Roman
Mesolithic	1.000	-	-	-	-	-
Neolithic	1.000	1.000	-	-	-	-
Copper Age	1.000	1.000	1.000	-	-	-
Bronze Age	<.001	<.001	<.001	<.001	-	-
Roman	0.785	0.650	<.001	<.001	<.001	-
Medieval	0.036	0.036	<.001	<.001	<.001	0.006
**ANOVA results**	df	F	Sig.			
	6	58.9472	<.001			

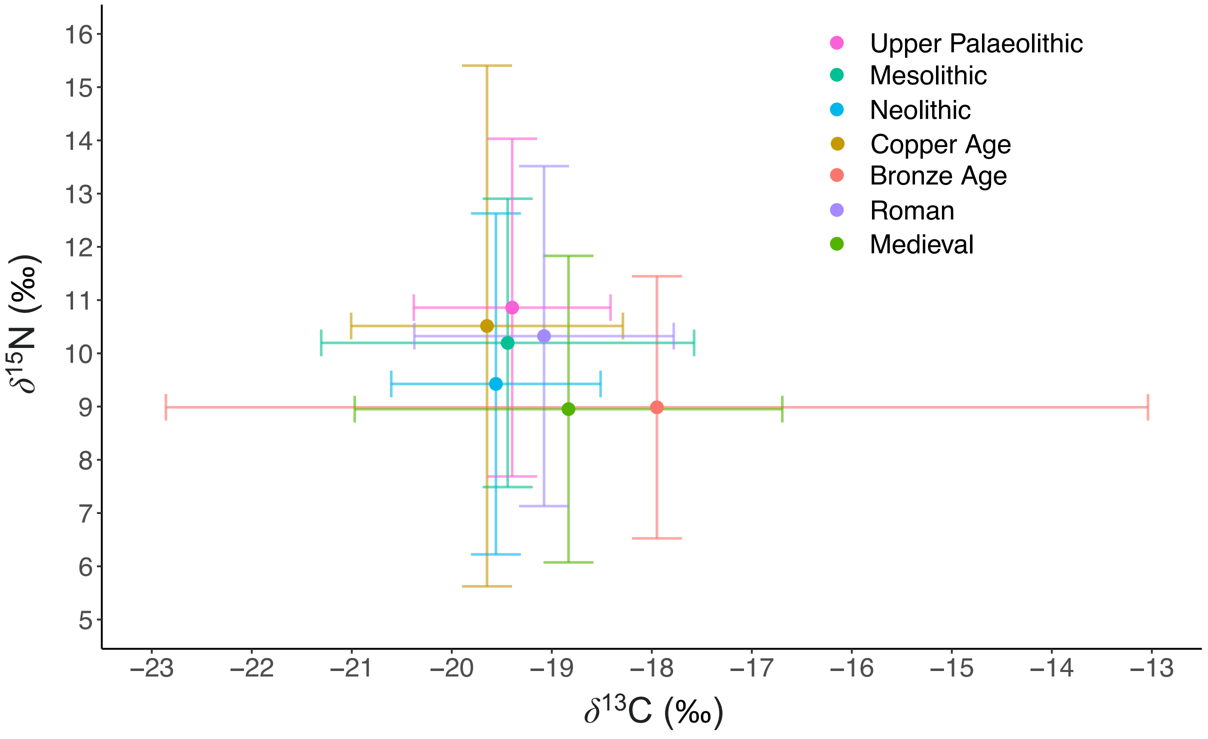

Figure S11 - Summary plot of $\delta^{13}C$ and $\delta^{15}N$ isotopes for 1986 human individuals from the central Mediterranean by traditional periods.

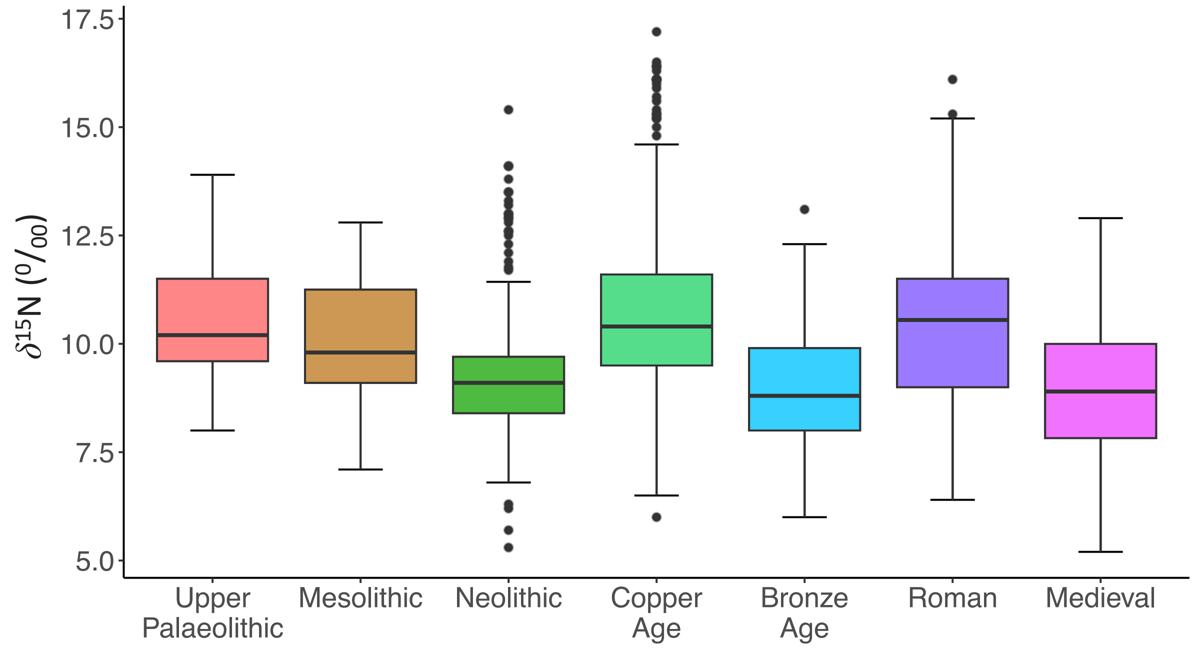

Figure S12: Nitrogen 15 (δ^{15}N) stable isotopes by period.

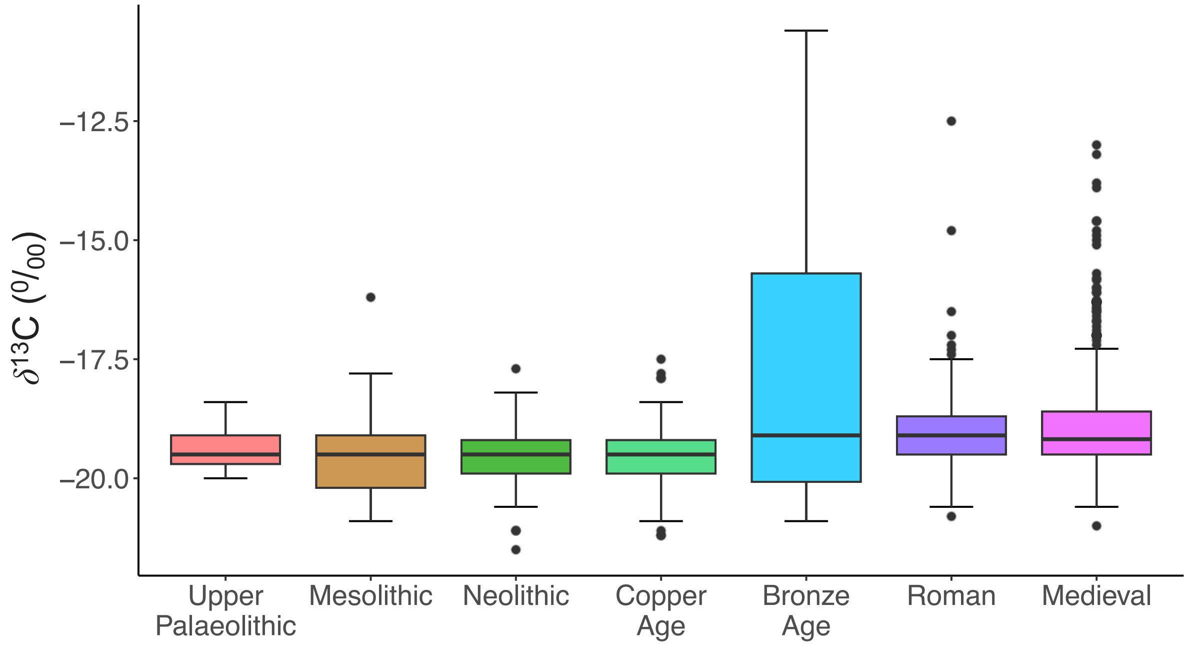

Figure S13: Carbon 13 (δ^{13}C) stable isotopes by period.

Section 5 – Statistical Analysis and *R* Code for LOESS plots

The following presents example code *R* code for generating LOESS plots and boxplots, in addition to details on statistical analysis provided in the Methods section of the main paper. Protocol for replicating KDE analysis is available in Supplementary Information for Parkinson *et al.* (2021)[4].

```
#Load relevant packages
Library(ggplot2)
Library(scales)

#LOESS scatter plots to explore continuous temporal trends in body mass
ggplot(DATA,
      aes(x = YearsBP, #Years BP of datapoint
          y = BM, #Body mass
          col = Time_Period)) + #Colour points by time period
  geom_smooth(method = "loess", color = 'black', fill = 'turquoise', span = .75, size = 1) +
#span is smoothing of loess line
  geom_point(size = 0.8) +  #change point size and colour
  ylab('Body mass (kg)') +
  xlab('Years BP') +
  scale_x_reverse(limits=c(30000,0), breaks = seq (30000, 0, by = -5000)) + #reverse scale
  ggtitle('Temporal Trends in Estimated Body Mass') +
  theme(axis.text=element_text(size=12), #change font size of axis text
       axis.title=element_text(size=15), #change font size of axis titles
       title =element_text(size=20), #change font size of title text
       legend.title = element_text(size = 20),
       legend.text = element_text(size = 15))

#Example box plots to explore temporal trends in body mass between individual time periods
ggplot(DATA,
 aes(`Time_Period`, BM, fill = `Time_Period`)) +
  geom_boxplot(
   col = "black", #outline colour
   alpha = 0.4, #transparency, 0-1
   notch = FALSE, #notch in boxplot
   notchwidth = 0.5, #size of notch, 1-0
   outlier.colour = "black", #colour of outlier
   outlier.shape = 1, #shape of outlier
   outlier.alpha = 0.3, #outleir transparency 0-1
   outlier.size = 2) + #size of outlier, put to NA to not show
  labs(            #Labels
   y = "Body mass (kg)",
   x = "Time Period",) +
  scale_x_discrete(labels = wrap_format(10)) +
  stat_boxplot(geom='errorbar', width = 0.3) + geom_boxplot(inherit.aes = TRUE) + #whiskers
  theme_classic() +
  theme(legend.position="none") +
  theme(axis.text=element_text(size=15), #change font size of axis text
      axis.title=element_text(size=20) #change font size of axis titles
  )
```

References

1. Ruff, C. B. *Skeletal Variation and Adaptation in Europeans: Upper Paleolithic to the Twentieth Century*. (Wiley, 2018). doi:10.1002/9781118628430.
2. Sparacello, V. S. et al. Changing mobility patterns at the Pleistocene-Holocene transition. in *Paleolithic Italy. Advanced Studies on Early Human Adaptations in the Apennine Peninsula* (eds. Borgia, V. & Cristiani, E.) 357–396 (Sidestone Press, 2018).
3. Sparacello, V. S. *et al*. Human remains from Arma di Nasino (Liguria) provide novel insights into the paleoecology of early Holocene foragers in northwestern Italy. *Sci Rep* **13**, 16415 (2023).
4. Parkinson, E. W., McLaughlin, T. R., Esposito, C., Stoddart, S. & Malone, C. Radiocarbon Dated Trends and Central Mediterranean Prehistory. *Journal of World Prehistory* 2021 **34**, 317–379 (2021).
5. Vermeersch, P. M. Radiocarbon Palaeolithic Europe database: A regularly updated dataset of the radiometric data regarding the Palaeolithic of Europe, Siberia included. *Data Brief* **31**, 105793 (2020).

END OF DOCUMENT
